# The Development of a Mindfulness-Based Music Therapy (MBMT) Program for Women Receiving Adjuvant Chemotherapy for Breast Cancer

**DOI:** 10.3390/healthcare4030053

**Published:** 2016-08-09

**Authors:** Teresa Lesiuk

**Affiliations:** Frost School of Music, University of Miami, 5499 San Amaro Drive, N306, Coral Gables, FL 33146, USA; tlesiuk@miami.edu; Tel.: +1-305-284-3650

**Keywords:** music, music therapy, mindfulness, breast cancer, attention, mood

## Abstract

Problems with attention and symptom distress are common clinical features reported by women who receive adjuvant chemotherapy for breast cancer. Mindfulness practice significantly improves attention and mindfulness programs significantly reduce symptom distress in patients with cancer, and, more specifically, in women with breast cancer. Recently, a pilot investigation of a music therapy program, built on core attitudes of mindfulness practice, reported significant benefits of enhanced attention and decreased negative mood and fatigue in women with breast cancer. This paper delineates the design and development of the mindfulness-based music therapy (MBMT) program implemented in that pilot study and includes clients’ narrative journal responses. Conclusions and recommendations, including recommendation for further exploration of the function of music in mindfulness practice are provided.

## 1. Introduction

### 1.1. Music Therapy in Oncology

Music Therapy is the clinical and evidence-based use of music interventions to accomplish individualized goals within a therapeutic relationship by a credentialed professional who has completed an approved music therapy program (American Music Therapy Association, 2016).

Music Therapy is a valuable and evidence-based treatment for patients with cancer. For example, evidence of significantly decreased anxiety and depression [1], significantly improved quality-of-life [2], and shorter length of hospital stay [3], are reported via randomized control studies. Moreover, researchers elucidate the role of music therapy in cancer care support for patients, staff, and families and evidence of its effectiveness to decrease symptom distress [4], and, as well, espouse the patient factors that contribute to successful music therapy outcomes for symptom distress [5].

### 1.2. Music and Mindfulness for Women with Breast Cancer

Mindfulness is the practice of being fully aware of occurrences in the present moment. It excludes preoccupation with the future or rumination with the past [6]. The moment-to-moment engagement directs individuals away from an “automatic pilot” response or “reactive” mode that can cause stress and harm to self [7]. Moreover, the practice of mindfulness involves being aware and attentive to one’s form of thoughts as opposed to the content of one’s thoughts [8]. Further, developing a decentered field of awareness helps individuals tolerate thoughts that may be dysfunctional [6]. This consciousness applies to feelings and body sensations.

Problems with attention [9,10,11,12] and symptom distress [13] are common clinical features reported by women who receive adjuvant chemotherapy for breast cancer. Mindfulness significantly improves attention [14,15,16,17], and, as well, significantly reduces symptom distress in women with breast cancer [18,19,20].

A main stimulus of focus for mindfulness practice is often one’s breathing. While mindfulness practice usually calls for attention to one’s breath, other stimuli, including auditory stimuli can serve as a source of contemplation. Graham [21] provided a mindful-music and mindful-environmental sound listening guide for readers and recommended the exploration and investigation of preferred music stimuli as a source of focus for mindfulness practice. Music and mindfulness significantly enhanced attention in women with breast cancer [22], addressing common cognitive problems associated with chemotherapy treatments, alias “chemo-brain” [23,24,25].

### 1.3. Purpose and Background to the Study

The purpose of this paper is to delineate the development and implementation of a mindfulness-based music therapy (MBMT) for women receiving adjuvant chemotherapy for breast cancer. Full descriptions of music therapy interventions allow for accurate replications and understanding of outcomes. Detailed chronicling of interventions used in investigations is referred to as treatment fidelity in research reporting [26,27].

The author of this paper is a university researcher and a credentialed music therapist (alias music therapist board-certified, MT-BC). She developed, implemented, and investigated the impact of a music and mindfulness intervention for women with breast cancer [22]. While the intervention is referred to as mindfulness-based music therapy, it can alternatively be referred to as a music and mindfulness intervention within a music therapy context. The music therapy research community is currently discussing the role of the term *music therapy* in intervention titles [28]. However, for the purpose of this paper, the title of the intervention retains the full terminology, mindfulness-based music therapy (MBMT).

The author provides specifics of the session structure, sequences of activities, and scripted instructions. Of note, the scripts are a guide for facilitators and are meant to be fluid and should not be used verbatim. In addition to recounting the session structure, select narrative entries from homework journals are also provided. This MBMT program may contribute to healthcare programs, mindfulness-based practices, and, as well, music therapy literature and clinical practice.

Of further note, the MBMT presented here was tested for its effectiveness to improve attention and mood in women receiving adjuvant chemotherapy for breast cancer, and is reported elsewhere [22].

## 2. Participants and Location

Fifteen women, mean age of 53 years, with a diagnosis of breast cancer, stage I (n = 2), stage II (n = 6), stage III (n = 7), receiving adjuvant chemotherapy were recruited from a cancer treatment center in south Florida. Participants were excluded from the study if there was a prior cognitive impairment such as a head injury or history or psychosis. Session meeting times that were agreeable to each woman were scheduled for one-hour per week. Most sessions were held in a comfortable room at a university in southern Florida, while a few women received their sessions in a comfortable room at the cancer center. Most women attended the sessions alone, while a few were accompanied with a family member, partner or friend. On occasion, a nurse from the cancer center who was very interested in mindfulness and music for the women joined the sessions and contributed to the discussions.

## 3. The Development of the MBMT Program

The MBMT program development was strongly influenced by the author’s experience with a mindfulness program called the Mindfulness-based Stress Reduction (MBSR) Program. Prior to the study, the author took part in a MBSR program held as a continuing education program offered at a local hospital. The MBSR training included 2-h weekly sessions over eight weeks consisting of mindfulness exercises of walking, yoga, sitting meditation, awareness and practice of mindfulness attitudes, and 45-min of daily homework. The MBSR program was established in 1979 by Jon Kabat-Zinn, a professor of medicine and student of Buddhist meditation, for cancer patients at University of Massachusetts’ Medical School [29]. The full description and benefits of the MBSR program are beyond the scope of this paper. Readers are directed to Kabat-Zinn’s Center for Mindfulness as one possible resource [30] and to a helpful MBSR guide that addresses depression and anxiety [31].

The author chose four mindfulness attitudes from a possible seven attitudes, espoused by the MBSR program, and implemented them sequentially into a four-week MBMT program. The four attitudes chosen translated fluently to music exercises and metaphors. The first attitude, called *non-judging*, refers to the absence of evaluating and/or absence of reacting in a habitual way to stimuli, whether the stimuli is initiated from internal dialogue or sensations, or external events. The second attitude, called *beginner’s mind*, is the practice of perceiving newness in one’s environment, even when the environment is very familiar. It is a positive attitude in which one is willing to practice a sense of wonder and leave old practiced ways of perceiving, especially negative ones. The third attitude, called *suspending judgment*, while similar to the first attitude, emphasizes the willingness to try out new experiences and suspend one’s critical voice. This attitude was smoothly implemented in the MBMT program when the women were challenged to be open to trying new music experiences. The fourth attitude, called *acceptance and letting go*, is a mental practice of viewing one’s experience without fighting or striving to change one’s current state.

All of the attitudes are intended to enhance the practice of being in the moment—the core purpose of mindfulness. The role of music is to support the mindfulness attitudes, which in turn support moment-to-moment experience. Delineation of the roles of music is provided in each MBMT session description. Moreover, the underlying mechanism of MBMT is based on the psychological concept of decentering, described by Bishop [6] as awareness of distressing thoughts as “transient mental events rather than as reflection of the self or as necessarily accurate reflections on reality”. Logically following then, music promotes the development of decentering awareness.

## 4. An Overview of the MBMT Program

The women received four-weekly individual one-hour MBMT sessions along with assigned homework throughout the four weeks. The MBMT program, designed by the music therapist (researcher), was intended to enhance the women’s attention ability and to decrease symptom distress. The initial music exercises required relatively passive participation from the women, but required greater active participation with each subsequent session. The fourth and last session returned to more passive participation.

Music activities varied each week and were comprised of focused music listening and writing, exploring new instruments, singing, imitating rhythms and playing instruments in ensemble, and music-assisted relaxation. Weekly music sessions were designed to complement a different mindfulness attitude for each of the four weeks (see Table 1). Every session began with an opening exercise, followed by a music experience coupled with a new mindfulness attitude. At the closing of each session, homework assignments, based on the in-session experience, were given. Daily homework practice of about 15 to 20 min was recommended. Pink folders (the color pink being symbolic for support of breast cancer) containing homework instruction sheets and weekly handouts, were provided to each woman. Four author-designed compact discs were also provided. Homework and between-session experiences of the mindfulness attitudes in daily life were discussed at the beginning of each subsequent session.

## 5. Opening Exercise with Focus on Sound

An opening exercise of approximately 5–7 min was developed and implemented weekly for the purpose of focusing the women’s attention to sound. The music therapist sounded out a single ring of a tone chime or thumb cymbal until there was no longer any sound. The women listened to the single ringing tone and indicated, by raising their hand, when they no longer heard any sound. A second focusing experience consisted of listening to a longer live improvisation on one of: piano, guitar, or xylophone. The music therapist led the women into the practice of mindfulness by speaking slowly and giving time between statements.

Script for opening exercise focus on sound:

In this exercise we will begin the practice of mindfulness. First, bring yourself into the present moment by sitting up straight, but comfortably. If possible, close your eyes lightly and take time to breathe deeply. Then ask yourself silently, “What is my experience right now…in bodily sensations or tensions (scan your body from your feet to your head)…in thoughts…in feelings?” Take time to acknowledge and register your experience, even if it is unwanted.

Now, gently redirect your attention to the sound of this bell and when you no longer hear it ring out—raise your hand … (sound of tone chime). Now, listen again, and listen to whether the sound is shorter or longer than what you just heard (play sound of tone chime). Was it shorter or longer? Now, again, listen to this … (play soft) …. And then listen to this … (play loud) … Which was louder?

A few minutes discussion of the women’s awareness of thoughts, feelings or body sensations ensued. Most often the women focused solely on the sound of the tone chime without distraction. The discussion of the sensations and sound of the bell reinforced the women being present with sound, vibration, and their resulting physical sensations evoked by the sound. After a brief discussion, the next auditory experience was introduced, again followed by discussion.

Script for opening exercise focus on music improvisation:

Continue to direct your attention to the music tones as they sound one after another. When your mind wanders and gets caught up in thoughts or feelings or other sensations, simply notice where your mind is, and gently bring your attention back to the music as best you can. Be aware that you do not have to follow your upcoming thoughts or feelings and try not to judge yourself for having them or analyze them in any way. It’s okay for the thoughts or feelings to be there. The music you hear can function as an anchor to bring you into the present and help you tune into a state of awareness of the present. Simply observe any thoughts or feelings, let them drift on by, and bring your attention back to the tones. (Music therapist improvises a short melodic or tonal sequence.)

### 5.1. Week One—Music Listening and Non-Judging

In the first session (Week One), a music listening exercise was used to introduce the practice of *non-judging*, a core mindfulness attitude. The music listening exercise served as a support to deepen listening skills and judgments of the music, and, more specifically, introduce the mindfulness attitude of *non-judging*. Music listening was chosen for the first MBMT activity because listening to music is a familiar music experience for most people as compared to playing an instrument. The music selections consisted of different styles of music of a contrasting nature.

The women listened to five different selections of music (see Supplementary A for the first five selections), played for no more than two minutes each. After each music selection a written response to several questions was required. The first music listening exercise was processed aloud with the music therapist modeling the possible type of responses. Leading questions were provided, such as: Did you like the music selection? What music elements did you hear? What was the emotion of the music? What did you notice in yourself—in your body, any images come to mind, any judgments about self?

Script for music listening exercise:

This is a music listening exercise in which you will hear a few selections of different styles of music and you are asked to identify, as best you can, what you hear in the music. You are also asked to write down your responses and then you will have time to share. Here is a listening chart that will guide you through the exercise.

After hearing each music selection, and following the columns on the chart, write down your “first impression” of the music selection (e.g., like/dislike, good/bad, etc.), then the music elements that stand out to you (e.g., voice, guitar, drum, fast rhythms, quiet volume), the emotion of the music (e.g., sad, happy, excited, etc.), and then lastly, anything you notice in yourself (memory, image, body sensations, or any judgments you have of yourself, others, the activity).

Further discussion ensued that linked the music listening experience to the non-musical experience, that of practicing the mindfulness attitude of non-judging.

Script for music listening follow-up discussion:

What was your experience with this exercise? Was this listening exercise a pleasant event or experience for you? Most music experiences, when chosen by a person, are positive experiences. (However, listening to a loud pounding bass rhythm coming from another driver might not be!) What were you aware of as you listened? Did your mind wander? If so, where did it go? Were you able to bring it back to the music? What, if any, judgments arose you took part in the music listening? Were you able to notice them and let them just be there, as opposed to reacting to them?

#### Homework of Non-Judging in Daily Living

Pleasant experiences are common in preferred-music experiences, while both pleasant and unpleasant experiences are the reality of daily living, and particularly for those undergoing treatment for breast cancer. The homework for week one consisted of writing daily about pleasant and unpleasant events. Similar to the deep focus required in the music listening exercise, the women were to observe their feelings, body sensations, and/or emotions that arose during those events. A structured chart requiring a written response was provided in which participants wrote about daily events, reactions in feelings and thoughts that arose, and, as they practiced non-judging, how their response changed, if at all.

The recommendation was to listen to a researcher-developed music CD for 15–20 min daily, along with completing the music listening chart, and, as well, process and write about at least one pleasant or unpleasant event daily.

Script for non-judgment homework:

This week, take note of events that are experienced as pleasant and those events that are unpleasant. Observe your thoughts, feelings, sensations and/or emotions that you become aware of during or after these events. Be attentive to the response produced and practice quickly letting go of your own biases and fears [31]. Non-judgment attitudes in mindfulness practice are an awareness quality nurtured by assuming a detached observance within your own experience. In this attitude, we are bystanders throughout our own experiences. Use the pleasant/unpleasant chart to guide your recording of at least one event daily.

See Supplementary A for Week One homework instructions, the music listening chart, the music playlist, and the non-musical mindfulness practice.

Select quote from woman’s homework of non-judging:

It was 4 a.m. and I couldn’t sleep … My mind teemed with little worries and then I conjured up a big one: A grizzly bear was tearing me apart and eating me, starting with my arm. I thought: Here’s an unpleasant one for the log. I felt tense and hot (chemo has restored my hot flashes). I was, in part, a detached observer … I decided to try and remember the music that had been part of the homework CD: Rodrigo tickling the Spanish guitar, [the opera singer] belting out the gorgeous area. It seemed to work: next thing I knew it was a respectable 7:30. I arose, took a hot shower, and felt like a new woman. Still feel that way hours later.

### 5.2. Week Two—Novel and Familiar Instruments/Songs and Beginner’s Mind

The second session (Week Two) consisted of novel instrument playing and singing familiar songs, both activities used to introduce the practice of *beginner’s mind*, a core mindfulness attitude. Instrument playing involved exploring the sound and touch of novel instruments such as a *mbira* or thumb piano, an ocean drum, pentatonic bars, and a rain stick. Exploring the feel and sound of novel instruments was designed to evoke an experience of wonder and pleasant surprise that is found in the attitude of *beginner’s mind*. Participants handled and produced sound on the novel instruments and spoke about their liking, sensory responses, and perceptions of their experience. Immediately following the novel instrument exploration the music therapist introduced the attitude of *beginner’s mind*.

Script for novel instrument playing and beginner’s mind exercise:

You will explore a few instruments that you may not have heard or seen before. Take time with each instrument and listen to its sound (what you are hearing)—be aware of what you feel (in your hands, on your lap, its vibrations). Now as you explore these instruments take time to notice your thoughts, feelings, and body sensations. Were you surprised or in awe of the sounds or feel of the instruments?

One attitudinal foundation of mindfulness practice is called beginner’s mind. It is an attitude of mind of practicing seeing (or hearing) the richness of the present-moment experience. Practicing the mindfulness attitude of beginner’s mind develops perceiving the world with a quality of newness and awe. This attitude is developed by seeing or experiencing events, things, and/or people with a child-like sense of wonder.

Following the novel instrument exercise, the women were then given a songbook that contained several well-known American songs, and, as well, individual preferred song requests were included as well. Examples of songbook titles included *Amazing Grace (from UK), Blowin’ in the Wind, Both Sides Now, Bridge Over Troubled Waters*, *Country Roads*, *I Can see Clearly Now, Let it Be*, and *You’ve Got a Friend*. Sample individual song title requests included *Fire and Rain*, and *Someone Like You.* The women were encouraged to sing along with the music therapist while she sang and accompanied the singing on piano or guitar. The women could also simply listen. While the song singing was enjoyable, the challenge of the exercise was to practice beginner’s mind with familiar music and hear or perceive something new in the music.

Script for beginner’s mind follow-up discussion:

Were you aware of anything new in your experience of singing or hearing these songs? What occurred in your thinking, feeling, body sensations? Did you have any judgments of the music or yourself?

Often, we let our beliefs about what we “know” prevent us from seeing things as they really are. To see the richness of the present moment, we need to cultivate this beginner’s mind attitude or a mind that is willing to see everything as if for the first time.

The music exercise and discussion served as a practice trial to see familiar daily environments, events, home, spouse, family and/or friends in a new or different way.

#### Homework of Beginner’s Mind in Daily Living

The women were to continue with daily practice of *beginner’s mind* using familiar music of either their own or using the music CD provided. The CD consisted of several different instrument sounds followed by several different music selections, some of which were piano performances by the music therapist. The women were also to continue practice of this attitude practice with daily events. Homework charts for both the music exercise and recording daily events were provided. A suggestion was given to listen to music CD and/or sing the familiar songs for 15–20 min daily and write about at least one non-music event daily utilizing beginner’s mind.

Script for beginner’s mind homework:

Where can you practice beginner’s mind today? Is there a time when beginner’s mind might be useful? Is there something you might view differently or anew? Is there something you might hear differently or anew? Someone you might see differently for the first time? Next time you see your friend, try to see something new in him or her. As you practice this attitude, it is helpful to consciously let go of past experiences and expectations. Witness the fullness of your new awareness. Use the beginner’s mind logs to record this week’s experiences to familiar music listening and to daily events/people. I look forward to hearing your experiences, whatever you experience, when you return next week.

See Supplementary B for Week Two homework instructions, the music listening exercise, and the non-musical mindfulness practice.

Select quotes from women’s homework of beginner’s mind:

About music listening:

Piano very joyful and upbeat, beautiful melody. I feel very calm and happy listening.

About daily living:

Plastic surgeon—I was afraid he wouldn’t listen, but he did, and embraced my plan for surgery. He’s no diva after all.

Sat outside in my yard quietly enjoying the plants and flowers without focusing on the “jobs” to be done—just enjoyed the air, trees, fragrance and peaceful environment. It was so relaxing.

### 5.3. Week Three—Rhythm/Instrument Playing and Suspending Judgment

In the third session (Week Three), several music instrument-playing activities were developed for the purpose of requiring sustained focus. The intention of the somewhat challenging session activities was to counteract the negative effects of “chemo-brain”. The session consisted of imitating and creating simple rhythms on a large paddle drum, playing simple harmonic changes on a xylophone, playing a bordun (a repeated pattern) on a piano keyboard, and playing a familiar melody by number on the keyboard. Dachinger [32] designed the latter two music exercises to improve impulsivity in adults with substance dependence. Women were given an egg shaker, an instrument shaped like an egg that sounds like a small maraca, to retain for their rhythm practice at home. The instrument playing exercises were also used to introduce the practice of *suspending judgment*, a core mindfulness attitude.

Script for rhythm and instrument playing:

Rhythmic imitation:

Let’s play some rhythms. This is a paddle drum (demonstrate sound using a mallet). I will play a rhythm on my drum and once I finish, you play it on your drum.

Playing simple harmonic changes on a xylophone:

Let’s play and sing a song together. Here is a song sheet that shows changes in harmony indicated by changes in color placed over the text. You will play this pulse or beat with these two mallets on these tones (marked with matching color to the text) for each harmony change. (Demonstrate beat and practice harmonic changes with an accompanying support, such as keyboard or guitar). Now, let’s play and sing this together. (If a family member or friend is in the session, she can play the drum on the chorus).

Playing a simple repeated pattern on the piano keyboard:

Here are two tones that you play together, called a bordun. Play at this tempo. (Demonstrate). Keep playing that pattern while I play a melody over top. (Music therapist improvises a melody above the bordun).

Playing a melody by number:

Notice where the numbers are labeled on the keyboard. Play from number 1 to number … (6). Follow the song sheet reading the numbers above the words and once you are comfortable feel free to play and sing along.

These music experiences served as a practice trial of the attitude to suspend judgment of self, especially when trying something new. The women were asked to share about their experience of their music instrument playing.

Script for suspending judgment discussion:

What was your experience with this activity? What, if any, judgments arose when you took part in the music making? Were you able to notice these judgments, observe them and let them go? Can you be aware of feelings, thoughts, judgment and not react to them? Just observe.

#### Homework for Suspending Judgment in Daily Living

The women continued with daily music practice with the use of a music CD provided to them. The researcher-designed CD consisted of 20 different simple rhythms and several rhythmic recordings of pop and rock music. The women used the egg shaker given to them and practiced imitating the rhythms they heard on the recording. Following the rhythm practice, the women kept beat to recorded rhythmic songs while enjoying the experience. They were also to practice the attitude of suspending judgment with daily events. Homework charts for both the music and daily events were provided. Daily 15–20 min practice with the music exercise was suggested and to write about at least one non-musical event daily.

Script for suspending judgment homework:

Practice with the music CD throughout the week. Try to practice imitating the rhythm items (try 4 to 5 rhythms per day), as heard on the CD, repeating the exercise as often as you’d like. Once you are comfortable with playing the rhythms, feel free to create your own rhythms!

Next, using your egg shaker, practice to keep beat to the recorded music on the CD as often as you’d like.

The next time you find your mind saying things like “I can’t do this”, “This won’t work”, “This is boring”, “I don’t like this”, remind yourself that the mindfulness practice involves suspending judgment and simply observing whatever comes up for you, including your judging thoughts, without pursuing them or acting on them. If you doubt this description of your mind, just observe how much you are preoccupied with liking and disliking during a ten-minute period as you go about your day. Record anything you might have done differently or tried that was new. What was your experience and what came up in your thoughts, feelings, or body sensations? Were you able to suspend judgment? (These instructions provided from MBSR)

See Supplementary C for Week Three homework instructions, an example of a song with harmonic color-coding, and the non-musical mindfulness practice.

Select quotes from women’s homework of suspending judgment:

Listened to a conversation where people gossiped about someone and I was starting to have negative judging thoughts about the person. I noticed [this] and stopped myself by thinking about something else.

Several issues come up for my [family member] and because of his narcissistic history I immediately didn’t believe him [or] the situation. I did stop that thought, listened to the story. For first time I did not get angry or frustrated, just tried to observe as an uninvolved party. I was more calm and detached, which was wonderful.

### 5.4. Week Four—Music-Assisted Relaxation and Acceptance/Letting Go

In the fourth session (Week Four), music-assisted relaxation was used to introduce the practice of *acceptance and letting go*, core mindfulness attitudes. The music therapist introduced deep breathing prior to the music-assisted relaxation exercise. A demonstration by the music therapist of deep, slow breaths that expanded the ribs was followed by the women practicing the same.

The music and scripted imagery served to facilitate relaxation and support visualization of letting go of concerns and distressing symptoms, and, as well, promote loving images and feelings. The women were informed that they would be led through a spoken and music-assisted relaxation and imagery exercise. They were asked to gently direct and maintain their full attention to the sound of the speaker’s voice. They were also told that if their mind wandered and was caught up in thoughts or feelings or other sensations, to simply notice their thoughts, and gently bring their attention back to the speaker’s voice as best they could.

The women were instructed to become observant of thoughts, feelings, and/or body sensations that they would like to have a respite from, such as being tired, worried, and so forth. They were also informed that as part of the imagery experience they would have the opportunity to send those issues away, at least for a little time. The music therapist then provided a relaxing improvisation on piano while speaking the script called *Sending Thoughts Away on Clouds*, described below. The women visualized placing their troublesome issues on images of clouds that drifted by in the sky. When in the script, *the clouds* (carrying issues) *drifted by*, the piano improvisation continued to support the imagery, without any speaking.

Script for music-assisted relaxation:

If your mind wanders and gets caught up in thoughts or feelings or other sensations, simply notice where your mind is, and gently bring your attention back to the speaker’s voice as best you can. It’s okay for the thoughts of feelings to be there. Just notice any feelings or body sensations, let them drift on by, and bring your attention back to the voice.

Imagery script No. 1 for music-assisted relaxation (“…” refers to time between statements)

Start by listening to the piano tones … Simply listen to the music and notice the sensations of your body as you listen to the music …Try to focus all of your attention on the speaker’s voice … (music continues) … As you listen, close your eyes and imagine that you are laying down on a lawn of soft grass, looking up at a blue sky … Let your mind become as clear and empty as a perfect blue sky. If you need a break from being tired or from worrying, or if any thoughts drift into your mind, imagine yourself breathing them out so that each thought forms a cloud that you send blowing across the clear, blue sky … Allow your thoughts to drift away from you, like clouds across the sky, until your mind becomes empty again, only filled by the image of a clear, blue sky … Continue with this visualization until you no longer hear the music.

A brief discussion of the women’s experience with the imagery followed. Many women shared the images that they sent away and the respite they encountered. They also spoke of their minds wandering during the relaxation and bringing their attention back to the speaker’s voice.

Imagery script No. 2 for music-assisted relaxation (“…” refers to time between statements)

The women were then asked to bring to mind a favorite place of comfort or relaxation. A second scripted imagery followed (Inner Smile Meditation by Tom Price, Michigan musician) read by the music therapist and accompanied by recorded music (Daniel Kobialka’s instrumental music *Love will Never Go Away* from his album *Beyond Embracing Dreams*).

We will continue to take some time to relax the body … Find a relaxed position, so you can be as comfortable as possible. Free your mind and your body of any stress and tension. Let go of draining thoughts and daily responsibilities, and just focus on this moment and my voice. Feel free to lightly close your eyes and be aware of your breathing, take deep, slow breaths … inhaling … and exhaling slowly (pause) … As you exhale, release all of the tension in your body and feel yourself start to relax.

Be aware of your body ... relax your head, release the tension from your shoulders, move any way you like to help you release tension. Feel your hands resting on your chair or lap … Let your legs sink into the chair, and feel your feet in a relaxed, flexed position. Be aware of the feeling and relaxation throughout your body … And the complete release of tension and stress …

Now, imagine a time in your life when you were in a joyous, relaxed state. For many people, it is a beautiful spot visited on vacation … but the beauty of this is, you may choose whatever your ideal place is … now visualize yourself in that place, wherever it is. It may be walking along a beach, amongst some beautiful trees, or a mountain hike, or it may be in a comfortable chair in your backyard … where it is, be there now while you the music plays … [Allow the music to sound for a few minutes here].

Now with all of your senses, breathe in the fresh air, feel the warm sun and the gentle breezes on your face. Internalize how this makes you feel. Does it make you smile? These are precious moments we have in our memory, and we can revisit them any time we wish.

Now bring this feeling, this smiling feeling—to the core of your being. Start with feeling the smile on your face, then move it in. Slowly move this smile into your body. Let it penetrate from inside your heart to the rest of your body. Feel this smile in every cell of your being.

Know now that you may return to this feeling anytime you wish. [Allow the music to sound for a few minutes here and then fade music here.]

Now slowly begin to become aware of the presence of those around you, the sounds of the room, the feeling of air on your skin. Feel your feet planted firmly on the ground and as you breathe deeply again, slowly open your eyes and become aware of your surroundings. [Stop music]

A discussion of the women’s experience with the music-assisted relaxation followed. The women shared imagery about their favorite places and the feelings that arose during the music-assisted relaxation. Acceptance of feelings and being present to the speaker’s voice was also discussed.

#### Homework for Acceptance/Letting Go in Daily Living

The women were given a compact disc of seven music-assisted relaxation scripts and were asked to practice with the recording for 15–20 min daily. They were also to practice the mindfulness attitude of “letting go” during the relaxation. To practice “acceptance” the women were asked to read a poem called “The Guest House” by Rumi and to be open to accept all emotional experiences. Journaling of experiences was encouraged.

See Supplementary D for Week Four homework instructions, the music journal exercise, and “The Guest” poem.

Select quotes from women’s homework of acceptance and letting go:

This week the music was extremely relaxing and beautiful. From the beginning of the CD my mood, thoughts and feelings changed. I was apprehensive, nervous, etc … Now I feel acceptance, relaxed, ready for the approaching surgery Totally with a positive attitude!

The air around the room was cool but once the music stopped the room felt really warm and hot. I imagined being so small that I could fit inside a flower. There were pleasant images.

## 6. Conclusions and Recommendations

The design and development of the MBMT program were influenced by mindfulness attitudes inherent in the mindfulness-based stress reduction programs [24]. Four mindfulness attitudes were introduced to the women and practiced through four-weekly corresponding music exercises that were facilitated by a music therapist. The attitudes were also transferred to non-music daily living exercises to be practiced at home. Weekly narrative responses from the women reflect an improvement in mood and provide an understanding of how the women benefit in change of perspective from the MBMT homework. A positive transformation in many women became apparent over time of the four-week program. Self-identities seemed to be communicated with strength over time and the women appeared more relaxed over time. The narrative comments illustrate this positive change. Further, MBMT was effective in significantly reducing negative mood states, especially fatigue, and increasing energy and, as well, significantly improved attention, as reported elsewhere [22].

This program emphasized four mindfulness attitudes of mindfulness that were simulated or practiced within a musical context, followed by practice in daily life. While present-moment practice with sound opened each session, there was not an extended time period of moment-to-moment mindfulness practice. The author felt the shorter time periods were more appropriate than longer durations as many of the women were new to mindfulness and many were coping with anxiety as a result of their cancer diagnosis. The author was aware that enhanced awareness of the moment could potentially exacerbate the anxiety. Deep breathing was always part of the opening exercise, so relaxation was also an integral part of each session.

There are many ways in which the program can be extended and/or improved. For example, the author chose to emphasize four mindfulness attitudes, but others such as *patience* and *trust*, could be implemented into music simulations. These two attitudes are very helpful to women enduring series of chemotherapy treatments. Of note, the nature of the attitudes is that they overlap, in that, for example, *patience* is required when practicing *acceptance*.

The MBMT study was designed for a four-week period, but for some women the program ended before the women completed their chemotherapy treatments. In clinical practice, the program should be offered throughout the treatment regimen and, as well, offered to women post-treatment.

Further, MBMT should be held in a group format and outcomes be investigated. The social support from group members could further enhance the desired outcomes of improved mood states and attention. As well, the effect of MBMT on outcomes measures such as working memory, quality-of-life, and immune system response [33] are recommended.

Specific to music therapy research, patient factors that contribute to desired positive outcomes of MBMT, such as those referred to previously [5], should be investigated. Patient factors such as relationship with the therapist, level of relaxation, and level of mindfulness awareness could be investigated. Moreover, the effect and function of the music in MBMT should be understood. Recent studies have included examination of the impact of mindfulness preferred-music listening on relaxation [34]. Britton [35] designed a music mindfulness program that consisted of breathing with music, music listening, body scan to music, and movement to music. He outlined three ways in which music listening and playing instruments supports mindfulness practice: music as primary object of attention, music as facilitator, and music as a nonspecific memory cue. *Music as the primary object* is the practice of maintaining attention to the sounds of preferred music. Attention is anchored to a specific instrument, melody, voice, or overall sound, and if and when the mind wanders to thoughts, feelings, it is directed back to anchor to the sound. Next, music can evoke thoughts, feelings, and/or sensation and when attention is brought to these responses music is serving as *facilitator*. In other words, the primary object of attention becomes the response to the music, and thus the object of attention might become an emotional response, thought or body sensation as the mindfulness object. Last, music acts as a *nonspecific memory cue* when it supports exercises in mindfulness that include body awareness or movement. Music may serve to cue mindful-movement as for example, in Kabat Zinn’s [36] body-scan exercise. Britton’s conjecture of the role of music in mindfulness interventions is an important contribution to mindfulness practice, music psychology and music therapy practice.

Finally, the role of music and mindfulness in theoretical frameworks within a cancer-specific model [37] should be explored.

MBMT is a valuable intervention for women receiving adjuvant chemotherapy for breast cancer. In the words of one participant when asked at the end of the program about any particular moments that stood out to her in her MBMT experience…

It was for me, the culmination of the experience and what the study gave to me or reinforced for me, was my love of music…

And then when asked how she would explain mindfulness to a group of people or friends…

Getting rid of all the extraneous crap so that you can live in the moment. Depending on the age of the group I may or may not use the word “crap”! … And stopping to smell the flowers and even if it’s in the middle of a garbage dump, you are smelling the flowers. You definitely focus on the positive. In fact, [a project being asked of me lately] has been nothing but aggravation. And I finally [decided to say] “You know, this music therapy that I am concluding today has made me realize that I don’t want to do this project. I want to have positive experiences right now, which may sound selfish, and is more selfish than I like to be, but it’s necessary right now.

## Figures and Tables

**Table 1:** Mindfulness-Based Music Therapy (MBMT) Weekly Session Structure.

Session	Music Experience	Mindfulness Attitude
1	Opening Exercise Music Listening and Writing	Non-judging
2	Opening Exercise Novel Instruments, Familiar Songs	Beginner’s Mind
3	Opening Exercise Rhythm, instrument playing	Suspending Judgment
4	Opening Exercise Music-assisted relaxation	Acceptance and Letting Go

## Supplementary Materials

Supplementary File 1
