# Supplementary material for: The Development of a Mindfulness-Based Music Therapy (MBMT) Program for Women Receiving Adjuvant Chemotherapy for Breast Cancer
Source: Healthcare (Basel). 2016 Aug 9;4(3):53. doi: 10.3390/healthcare4030053 (PMC5041054; doi:10.3390/healthcare4030053)
Supplement: Supplementary File 1 [file healthcare-04-00053-s001.docx]

Supplementary Materials: The Development of
a Mindfulness-Based Music Therapy (MBMT) Program for Women Receiving Adjuvant Chemotherapy for Breast Cancer

Teresa Lesiuk

**Supplementary A**

Supplementary A contains, in order, Week One homework instructions, the music listening chart, the music playlist, and the *non-judging* attitude daily exercise form.

*Week 1*

*Music Listening*

**Mindfulness Attitude—Non-judging in Pleasant/Unpleasant Events**

**15–20 Minutes a Day**

**The Music Listening Exercise**

Practice this music listening exercise throughout the week. Try to listen to 3 selections daily from the CD provided. You may listen to more if you would like and write as much as you want, repeating the exercise as often as you’d like.

You will hear selections of different styles of music and you are asked to identify, as best you can, what you hear in the music. You are also asked to write down your responses. Please use the listening chart to guide you through the exercise.

After hearing each music selection, and following the columns on the chart, write down your “first impression” of the music selection (e.g., like/dislike, good/bad, etc.), then the music elements that stand out to you (e.g., voice, guitar, drum, fast rhythms, quiet volume), the emotion of the music, and then lastly, anything you notice in yourself (memory, image, body sensation, any judgments).

**The Mindfulness Attitude Exercise**

Using the pleasant/unpleasant chart, note throughout the week (perhaps once daily) a pleasant or unpleasant experience. Whatever the experience, take a moment to observe it in a detached manner, taking note of what comes to mind or to your feelings. Practice being aware of any judgments you might have, but not reacting to those judgments. Be aware, like a bystander, how often you are preoccupied with or liking or disliking what you are feeling. Journal about the events and your observations.

**MBMT**

**A Music Listening Exercise**

| **Your First Impression**  (e.g., like or dislike, good or bad) | **Music Elements**  (e.g., instruments, fast or slow tempo, the texture, quiet or loud) | **Emotion of the Music**  (e.g., sounds peaceful, sad, excited) | **Anything You Notice in Yourself** (e.g., memories, images, body sensations, any judgments) |
| --- | --- | --- | --- |
|  |  |  |  |
|  |  |  |  |
|  |  |  |  |
|  |  |  |  |
|  |  |  |  |
|  |  |  |  |
|  |  |  |  |

**Music Playlist for Week One Homework**

**(Chosen from the Author’s Music Library)**

**(2 Min Each)**

| **Composer/Artist** | **Title** | **Style** |
| --- | --- | --- |
| 1. Copland | Rodeo | Classical |
| 2. Santana | Smooth | Rock |
| 3. Kelly Sweet | Caresse Sur Ocean | New Age |
| 4. Grits | Ooh Aah | Hip-Hop |
| 5. Whitacre | Sleep | Choral |
| 6. Elgar | “Nimrod” from Enigma Variations | Classical |
| 7. Rodrigo | 2nd mov., Concierto de Aranjuez | Classical |
| 8. Puccini | Nessun dorma—from Turandot (2.58) | Opera |
| 9. Samuel Barber’s | Adagio for Strings | Classical |
| 10. Pachelbel | Canon in D Major | Classical |
| 11. Jesse Cook | That’s Right from Vertigo | Nuevo Flamenco |
| 12. Copland | “Simple Gifts” from Appalachian Springs | Classical 5’37 |
| 13. Daniel Kolbialka | “Blooming in the Field” | New Age |
| 14. Paul McCartney | “Let it Be” | Classic Rock |
| 15. The Allman Brothers | “Stormy Monday” | Rock |
| 16. Ann Hamptom Calloway | “Finding Beauty” | Jazz |
| 17. Schubert/Horowitz | Impromptu Eb | Classical |
| 18. Santana | “Samba pa ‘Ti’” | Rock |
| 19. Thelonious Monk | Blue Monk from Monk’s Blues | Jazz |
| 20. Toot Tielman | “Bluesette” | Jazz |
| 21. Gypsy Kings | Djobi Djoba | World Music |
| 22. J. S. Bach | Herr, Deine Augen.. Cantata 102.. | Classical |
| 23. Barbara Streisand | Here’s that Rainy Day | Jazz-Pop |
| 24. Paul Simon | Bridge Over Troubled Waters | Pop |

**Pleasant/Unpleasant Events**

Please log at least one daily Pleasant/Unpleasant event.

| **What Was the Experience?** | **Were You Aware of the Pleasant/Unpleasant Feelings While the Event Was Happening?** | **How did Your Body Feel, in Detail during this Experience?** | **What Moods, Feelings and Thoughts Accompanied this Event?** | **What Thoughts Are in Your Mind Now as You Write About this Experience?** |
| --- | --- | --- | --- | --- |
|  |  |  |  |  |
|  |  |  |  |  |
|  |  |  |  |  |
|  |  |  |  |  |
|  |  |  |  |  |

**Supplementary** **B**

Supplementary B contains, in order, Week Two homework instructions, the music exercise form and the *beginner’s mind* attitude daily exercise form.

*Week 2*

*Song Selection*

**Mindfulness Attitude—Beginner’s Mind**

**Homework—15–20 Minutes a Day**

**A. Music**

Listen to sounds and music. What did you notice about the sounds/music? Were you aware of the length of sound, characteristic of the sound (e.g., nasal), fastness, high/lowness? Did you notice any response in yourself to the sound? Listen to one selection of music each day from the CD provided OR sing the songs from the songbook provided OR listen to music from your own music collection. Using the Beginner’s Mind MUSIC FORM provided—write down what you listened to and journal your experience.

**B. Environment**

Choose someone or something you know and intentionally look at this person/object with a ***Beginner’s Mind*** or fresh eyes. Are you seeing this person/object as they really are or are you only seeing the reflection of your own thoughts about them? Try this with a friend, significant other, children, and/or
co-workers. Try this with your body. Try this with nature (the sky, the stars, the ocean, and the trees). Journal about each new experience.

**Beginner’s Mind—Music And Sound**

| Listen to sounds and music. What did you notice about the sounds/music? Were you aware of the length of sound, characteristic of the sound (e.g., nasal), fastness, high/lowness? Did you notice any response in yourself to the sound? Listen to one selection of music each day from the CD provided OR sing the songs from the songbook provided OR listen to music from your own music collection. Write down what you listened to and journal about your experience. |
| --- |
|  |
|  |
|  |
|  |
|  |
|  |

**Beginner’s Mind—Environment**

| Choose someone or something you know and intentionally look at this person/object with a Beginner’s Mind or fresh eyes. Are you seeing this person/object as they really are or are you only seeing the reflection of your own thoughts about them? Try this with a friend, significant other, and/or co-workers. Try this with your body. Try this with nature (the sky, the stars, the ocean, and the trees). |
| --- |
|  |
|  |
|  |
|  |
|  |
|  |

**Supplementary C**

Supplementary C contains, in order, Week Three homework instructions, “Lion Sleeps Tonight” with color-coded harmony, and the *suspending judgment* attitude daily exercise form.

*Week 3 homework*

*Music Playing*

**Mindfulness Attitude—Suspending Judgment and Acceptance of Self.
Trying Less, *Becoming* More.**

**The Music Playing Exercise**

Practice with the music playing CD throughout the week. Try to practice the rhythm copying exercises (try 4 to 5 rhythms per day), as heard on the CD, repeating the exercise as often as you’d like. Once you are comfortable with playing the rhythms, feel free to create your own rhythms!

Using your egg shaker, try to keep beat to the recorded music on the CD as often as you’d like.

**The Mindfulness Attitude Exercise**

The next time you find your mind saying things like “I can’t do this”, “This won’t work”, “This is boring”, “I don’t like this”, remind yourself that the mindfulness practice involves suspending judgment and simply observing whatever comes up for you, including your judging thoughts, without pursuing them or acting on them.

If you doubt this description of your mind, just observe how much you are preoccupied with liking and disliking during a ten-minute period as you go about your business. Record anything you might have done differently or tried that was new. What was your experience and what came up in your thoughts, feelings, or body sensations? Were you able to suspend judgment?

**The Lion Sleeps Tonight**

| ***Chorus*** |
| --- |
| *I IV I V7* |
| Wee------------ ooh wim-o-weh |
| Wim-o-weh, o-wim-o-weh, o-wim-o-weh, o-wim-o-weh, o- |
| wim-o-weh, o-wim-o-weh, o-wim-o-weh, o-wim-o-weh |
| Verse I |
| In the jungle, the mighty jungle the, lion sleeps to-night |
| In the jungle, the mighty jungle the, lion sleeps to-night |
| Verse II |
| Near the village, the peaceful village, the lion sleeps to -night |
| Near the village, the quiet village, the lion sleeps to-night |
| Verse III |
| Hush my darling, don't fear my darling, the lion sleeps to-night |
| Hush my darling, don't fear my darling, the lion sleeps to-night |

Notes: Blue—I, Green—IV, Purple—V7.

**Suspending Judgement**

| Mindfulness practice involves suspending judgment and simply observing whatever comes up for you, including your judging thoughts, without pursuing them or acting on them. If you doubt this description of your mind, just observe how much you are preoccupied with liking and disliking during a ten-minute period as you go about your business. Record anything you might have done differently or tried that was new. What was your experience and what came up in your thoughts, feelings, or body sensations? Were you able to suspend judgment? |
| --- |
|  |
|  |
|  |
|  |
|  |
|  |
|  |

**Supplementary D**

Supplementary D contains, in order, Week Four homework instructions, the music exercise form and the poem “The Guest House”.

*Week 4 cd*

*Music and Imagery*

**Mindfulness Attitude—Letting Go and Acceptance**

**Homework Exercise**

1. Practice with the CD the music-imagery for 15 minutes daily. Also practice the mindfulness attitude of “letting go” to the relaxation.

2. Practice the mindfulness attitude of acceptance of your experiences. Read “The Guest” and practice acceptance of your emotional experiences throughout the week.

3. Accept the emotions, thoughts, body sensations that come to you as you experience the music-imagery. Journaling your experiences is encouraged.

**Letting Go**

Music and imagery—“Letting Go”

| Your mind may relax when you have released certain tensions, unwanted thoughts and worries. Using the homework CD practice the music-imagery for 15 minutes daily and the mindfulness attitude of “letting go”. What images were pleasant for you? Have your thoughts, feelings, or body sensations changed as a result of the deep listening? Try to remain focused on the speaker’s voice and then journal about your experiences. Also feel free to journal about non-musical experiences when you were able to “let go” during the week. |
| --- |
|  |
|  |
|  |
|  |
|  |

**The Guest House**

This being human is a guest house

Every morning a new arrival.

A joy, a depression, a meanness,

some momentary awareness comes

as an unexpected visitor.

Welcome and entertain them all!

Even if they’re a crowd of sorrows,

who violently sweep your house

empty of its furniture,

still, treat each guest honorably.

He may be clearing you out

for some new delight.

The dark thought, the shame, the malice,

meet them at the door laughing,

and invite them in.

Be grateful for whoever comes,

because each has been sent

as a guide from beyond.

Say I Am You poems of Rumi Translated by John Moyne and Coleman Barks

May 1994

©2016 by the author; licensee MDPI, Basel, Switzerland. This article is an open access article distributed under the terms and conditions of the Creative Commons Attribution (CC-BY) license (http://creativecommons.org/licenses/by/4.0/).
